# Supplementary material for: U.S. Physicians’ Training and Experience in Providing Trauma-Informed Care in Clinical Settings
Source: Int J Environ Res Public Health. 2024 Feb 16;21(2):232. doi: 10.3390/ijerph21020232 (PMC10888540; doi:10.3390/ijerph21020232)
Supplement: Supplementary file 1 [file ijerph-21-00232-s001.zip › ijerph-2848680-supplementary.pdf]

Supplementary Material

U.S. Physicians' Training and Experience in Providing Trauma-Informed Care in Clinical Settings

S1. Trauma-Informed Care Physician Assessment Tool (TIC-PAT)

Traumatic events such as sexual assault, domestic violence, childhood sexual abuse, exposure to combat, and elder abuse are common in the U.S. The following questions relate to your experience in providing care for those who have experienced traumatic events.

Patient-Centered Communication and Care

1. I ask what can be done to make patients more comfortable during the appointment.
   A. Always
   B. Often
   C. Sometimes
   D. Rarely
   E. Never
2. Prior to a physical examination, I present a brief summary of what parts of the body will be examined and allow the patient to ask questions.
   A. Always
   B. Often
   C. Sometimes
   D. Rarely
   E. Never
3. I give the option of shifting an item of clothing out of the way rather than putting on a gown when an entire area does not need to be examined.
   A. Always
   B. Often
   C. Sometimes
   D. Rarely
   E. Never
4. I offer patients a pillow for their back if they are anxious about being in the supine position.
   A. Always
   B. Often
   C. Sometimes
   D. Rarely
   E. Never
5. I give patients the option of a mirror for patients to see procedures or examinations that are out of their field of vision.
   A. Always
   B. Often
   C. Sometimes
   D. Rarely
   E. Never
6. I offer patients a way to "signal" anxiety verbally or nonverbally (e.g., raising their hand) during procedures/exams (e.g., Pap smear).
   A. Always
   B. Often
   C. Sometimes
   D. Rarely

E. Never

Understanding the Health Effects of Trauma

7. Select which of the following you would consider as possible indicators of trauma history in a patient.
    - Anxiety
    - Insomnia
    - Difficulty trusting others
    - High intake of sugary food and drink
    - Smoking
    - Drinking
    - Substance abuse (illicit or prescription)
    - Engaging in unprotected sex
    - Overeating
    - None of the above
8. I discuss with patients the relationship between unhealthy behaviors (maladaptive coping methods) and stress/trauma.
    A. Always
    B. Often
    C. Sometimes
    D. Rarely
    E. Never
9. When discussing behaviors that are detrimental to the patient's wellbeing, I brainstorm with them potential solutions for how to change the behavior.
    A. Always
    B. Often
    C. Sometimes
    D. Rarely
    E. Never

Interprofessional Collaboration

10. I maintain a list of referral sources for patients who disclose a trauma history.
    A. Strongly agree
    B. Agree
    C. Neither agree nor disagree
    D. Disagree
    E. Strongly disagree
11. I am confident in sensitively referring a patient with trauma.
    A. Strongly agree
12. Referral and educational materials about trauma for patients are readily available in the waiting room.
    A. Agree
    B. Neither agree nor disagree
    C. Disagree
    D. Strongly disagree
    E. Strongly agree
    F. Agree
    G. Neither agree nor disagree

H. Disagree
I. Strongly disagree

13. I am confident in working with nurses, medical interpreters, first responders, and others when caring for patients who have experienced trauma.
    A. Strongly agree
    B. Agree
    C. Neither agree nor disagree
    D. Disagree
    E. Strongly disagree

Understanding Personal Trauma History and Reactions

14. I reflect on my own stress and/or trauma history and how it may influence patient interactions.
    A. Strongly agree
    B. Agree
    C. Neither agree nor disagree
    D. Disagree
    E. Strongly disagree
15. I recognize when caring for patients with a trauma history begins to impact my own emotional health and wellbeing.
    A. Strongly agree
    B. Agree
    C. Neither agree nor disagree
    D. Disagree
    E. Strongly disagree
16. I practice self-care strategies (e.g. exercise, social support, etc.).
    A. Always
    B. Often
    C. Sometimes
    D. Rarely
    E. Never
17. I use counseling/mental health services to help me manage stress when I experience high levels of stress.
    A. Always
    B. Often
    C. Sometimes
    D. Rarely
    E. Never

Screening

18. I screen for trauma in every new patient.
    A. Always
    B. Often
    C. Sometimes
    D. Rarely
    E. Never
19. It is important to assess every case of trauma.
    A. Strongly agree
    B. Agree

    C. Neither agree nor disagree
    D. Disagree
    E. Strongly disagree
20. I screen for current trauma or a history of traumatic events as a follow-up to the universal screening questions.
    A. Always
    B. Often
    C. Sometimes
    D. Rarely
    E. Never
21. I (or my office staff) prepare a patient for potentially difficult questions prior to a trauma screening (e.g. reviewing confidentiality).
    A. Always
    B. Often
    C. Sometimes
    D. Rarely
    E. Never
22. Communication skills training is provided to all office staff about how to sensitively talk to patients who disclose a history of trauma.
    A. Always
    B. Very often
    C. Sometimes
    D. Rarely
    E. Never

About You

23. In your estimation, what percentage of your caseload has experienced a traumatic event (e.g. sexual trauma, interpersonal violence, community violence)?
    A. More than 50%
    B. 40-50%
    C. 30-40%
    D. Less than 30%
    E. Prefer not to answer
24. Indicate whether you have received training in the following: (Yes, No, Unsure)
    - Patient-centered communication and care
    - Understanding the health effects of trauma
    - Interprofessional collaboration
    - Understanding how your own history of stress/trauma may impact you professionally
    - Screening for traumatic events (with all new patients)
    - Screening for traumatic events in patients who are experiencing difficulties in current functioning (ex. Sleep difficulties, interpersonal problems, etc.)
    - Prefer not to answer
25. Indicate how much you agree the following inhibit your ability to practice trauma-informed care: (Strongly agree, Agree, Neither agree nor disagree, Disagree, Strongly disagree)
    - Interdisciplinary collaboration - lack of knowing the right provider to refer patient
    - Lack of time - scheduling conflicts, high patient load, short appointment time

- Access to resources - limited in-network services, waiting lists for services, lack of culturally and linguistically appropriate services, lack of geographically accessible services
- Difficulty managing your own levels of stress/practicing self-care
- Institutional constraints (ex. expectations for high patient load, paperwork)
- Your own trauma history (ex. lack of access to mental health support, feeling activated/triggered by trauma-related topics)
- Slow to implement change in the practice setting
- No clear process to establish trauma-informed care (ex. lack of institutional support)
- Educational institution did not highlight trauma-informed care
- Prefer not to answer

26. Gender
    - A. Female
    - B. Male
    - C. Transgender Female
    - D. Transgender Male
    - E. Gender Variant/Non-conforming
    - F. Not listed
        - ■ Open response
    - G. Prefer not to answer
27. Age
    - A. <35
    - B. 35-45
    - C. 46-55
    - D. 56-65
    - E. 66-75
    - F. 76+
    - G. Prefer not to answer
28. Race
    - A. Asian or Pacific Islander
    - B. Black or African American
    - C. Native American or Alaskan Native
    - D. White or Caucasian
    - E. Multiracial or Biracial
    - F. Other
    - G. Prefer not to answer
29. Ethnicity
    - A. Not of Hispanic, Latino, or Spanish origin
    - B. Yes, Mexican, Mexican Am., Chicano
    - C. Yes, Puerto Rican
    - D. Yes, Cuban
    - E. Yes, another Hispanic, Latino, or Spanish origin
        - ■ Indicate origin
    - F. Prefer not to answer
